# Virtual Reality Interventions and Chronic Pain: Scoping Review

**DOI:** 10.2196/59922

**Published:** 2025-02-18

**Authors:** Michael E Ding, Hajar Traiba, Hector R Perez

**Affiliations:** 1 Department of Medicine Division of General Internal Medicine Montefiore Medical Center Bronx, NY United States

**Keywords:** virtual reality, chronic pain, scoping review, pain management, efficacy, anxiety disorders, mood, health condition, health intervention, adults, aging, therapeutic, descriptive–analytical method, monitoring, US, PRISMA

## Abstract

**Background:**

Virtual reality (VR) interventions have demonstrated efficacy for more than a decade for mood and anxiety disorders and emerging evidence suggests they can reduce pain symptoms in both acute and chronic pain. More recently, these interventions have abounded within the commercial and academic sectors, immersing participants within a virtual environment to confer health benefits to users. VR immersion can facilitate the delivery of health interventions by isolating participants from distractors and stressors in a therapeutic environment. While recent studies of VR interventions have exploded, they are not uniform in approach or device type, limiting generalizability. Recent scoping reviews on VR and chronic pain have focused on specific diseases or limited inquiries to specific interventions or study types.

**Objective:**

We conducted a scoping review to generate new knowledge about the sum total of VR studies on chronic pain with specific emphasis on the methods and results of each study, including (1) the type of interventions, (2) outcomes chosen, (3) samples studied, and (4) data generated.

**Methods:**

A scoping review was performed on the literature on VR and chronic pain to describe themes associated with the literature to date and identify important gaps and unanswered questions to guide future research. CINAHL [EBSCO] (Cumulative Index to Nursing and Allied Health Literature) and PubMed were queried for the terms “virtual reality” and “pain,” providing studies of chronic pain adult participants using VR delivered through headset displays. We included English-language manuscripts that had at least one VR intervention arm with adults with chronic pain. For this analysis, we only included VR interventions that were immersive (ie, using headsets). Non–study reports, studies with no specific chronic pain component, those not involving adults, and those using VR as part of a comprehensive rehabilitation program were excluded. A descriptive analytical method was used to extract data, compare studies, and contextualize the presented outcomes. Articles were categorized into several themes including the type of intervention, outcomes chosen, participant characteristics, degree to which immersion was achieved, and adverse effect monitoring and reporting.

**Results:**

A total of 36 articles were included in our analysis. We summarize the literature using 5 themes: (1) heterogeneity of chronic pain types, (2) highly variable intervention types, (3) highly variable secondary and exploratory outcomes, (4) immersion was highly variable between studies and not systemically explored in many articles, and (5) side effect monitoring was limited.

**Conclusions:**

The literature on VR in chronic pain is highly variable and lacks theoretical rigor. While there is emerging evidence that supports VR use in a wide variety of health conditions including chronic pain, future research should focus on producing theoretically rigorous work that focuses on mechanisms and that systematically assesses side effects to generate robust generalizable knowledge.

## Introduction

Chronic pain affects 50 million US adults [1], and treatments have limited long-term efficacy, significant side effects, or limited accessibility [2-5]. As a result of the continuing opioid overdose crisis, numerous clinical and research guidelines have recommended further study of nonpharmacological options [6-8].

Virtual reality (VR) treatments have the potential to immerse participants within a virtual environment to confer health benefits to users. VR typically uses a headset to display a 3D image or video that participants can watch or interact with. Immersion produced through VR can facilitate the delivery of health interventions by isolating participants from distractors and stressors in a therapeutic environment [9]. VR interventions have demonstrated efficacy for more than a decade for mood and anxiety disorders [10,11], and emerging evidence suggests they can reduce pain symptoms in both acute and chronic pain [12-15].

Studies of VR interventions have exploded in recent years, due to advances in technology and the widespread availability of consumer-facing VR devices. Unfortunately, studies to date of VR in chronic pain have not been uniform in approach or device type, limiting generalizability. Recent scoping reviews on VR and chronic pain have focused on specific diseases or limited inquiries to specific interventions or study types [13,15]. We conducted a scoping review specifically to generate new knowledge about the sum total of VR studies on chronic pain with specific emphasis on the methods and results of each study, including (1) the type of interventions, (2) outcomes chosen, (3) samples studied, and (4) data generated. We expect the results of this scoping review will assist in generating new hypotheses and provide new areas of research inquiry for chronic pain VR researchers.

## Methods

We conducted a scoping review of the literature on VR and chronic pain to describe themes associated with the literature to date and identify important gaps and unanswered questions to guide future research.

### Eligibility Criteria

To develop the eligibility criteria, we conducted an initial search of the literature on PubMed and CINAHL and reviewed the first 100 articles that had initially resulted from general search terms of “virtual reality” and “pain.” As a first step, reviewers (HP, MD, and HT), independently screened these articles using Covidence software, followed by a meeting to discuss workflow and conflicts. Consensus was made regarding inclusion and exclusion criteria, which will be discussed below.

We decided to include English-language manuscripts and abstracts if at least one intervention arm included patients with chronic pain (even if compared with patients without chronic pain). We included patients with chronic pain of any type, even rare chronic diseases (eg, phantom limb syndrome). Because we noted some manuscripts defined VR as interchangeable with “interactive game” and because we only wished to explore studies about immersive VR, we only included studies of adult participants that used VR delivered through headset displays, not through other modalities (such as projection, computer screens, or television screens). We excluded nonstudy reports (letters, commentaries, and reviews), studies with patients with no specific chronic pain component (eg, acute burns), studies not involving adult patients, and studies using VR as part of a comprehensive rehabilitation program (eg, VR gait training). We did not focus on a particular set of outcomes of interest, and instead aimed to describe relevant outcomes in this research. We included research studies, case studies, and qualitative studies that met our inclusion criteria.

### Information Sources

**Figure 1:**
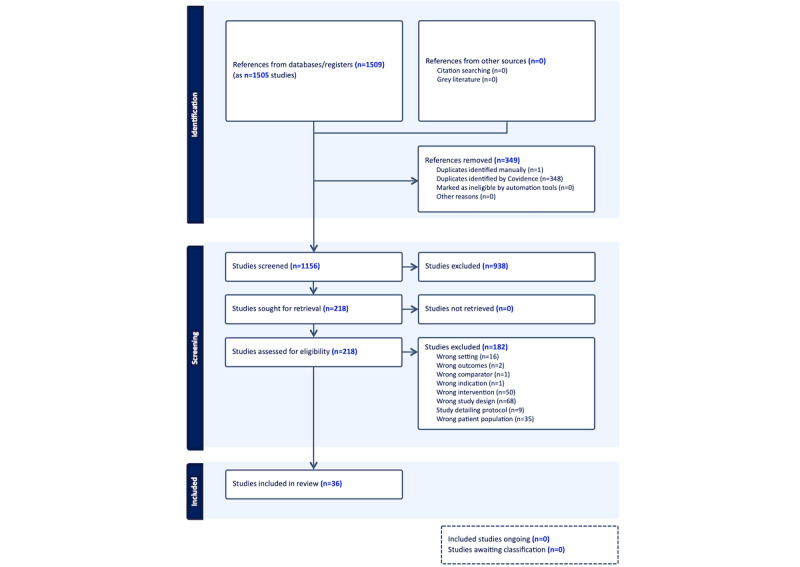
PRISMA (Preferred Reporting Items for Systematic Reviews and Meta-Analyses extension for Scoping Reviews) flowchart.

We performed a literature search of 2 electronic databases, CINAHL and PubMed between April 1, 2006, and March 21, 2022. These 2 comprehensive databases reference the nursing, allied health, and medical literature. Search terms included “Virtual reality” and “pain” to be broad and included all publications from the inception of both databases. We elected not to use MeSH (Medical Subject Headings) terms in searches to be sufficiently broad in our initial search. Results were prescreened using Covidence software (Melbourne, Victoria, Australia), with duplicate articles removed. The databases and platforms are outlined in the PRISMA-ScR (Preferred Reporting Items for Systematic Reviews and Meta-Analyses extension for Scoping Reviews) diagram (Figure 1).

### Selection of Sources of Evidence

After an initial prescreen, all citations and abstracts were screened by at least 2 independent reviewers (HP, MD, and HT). Abstracts that passed initial screening were included in a full-text eligibility review conducted by the same reviewers as above, with disagreements resolved by the senior author (HP).

### Data Coding and Synthesis of Results

We used a data abstraction form our team designed to extract data using Covidence software. The major categories on the data abstraction form included are (1) study characteristics (year of publication, authors, and country of origin), (2) research setting (population, study design, and research problem), (3) intervention (detailed description of intervention type and immersion quality), and (4) results and conclusions. Two reviewers (MD and HT) and 1 senior reviewer (HP) extracted data; HP resolved discrepancies. Subsequently, we used a descriptive-analytical method to synthesize data, compare studies, and contextualize the presented outcomes [16]. In our review, we categorized articles into several themes to illustrate the diversity of the literature. We focused on some specific elements a priori and allowed for additional themes to emerge upon our reading of the literature. Specific elements categorized included are (1) the type of intervention, (2) outcomes chosen, (3) participant characteristics, (4) the degree to which immersion was achieved, and (5) adverse event monitoring and reporting.

## Results

### Overview

After removing duplicate citations, we screened 1156 studies using titles and abstracts, and retrieved 218 articles for full-text screens for eligibility. We included 36 articles in the final sample [17-52]. About two-thirds (n=23, 63.9%) were completed in the United States [17,20-24,28,29,32-35,37-41,43,48-52]. About one-fifth (n=7, 19.4%) were randomized controlled trials [22-24,27,30,45,46], 6 (16.7%) were randomized cross-over studies [18,19,31,36,40,41], 16 (44.4%) were single-arm case studies, case series, or cohort studies [17,20,21,26,28,29,32-35,37-39,43,49,52], 6 (16.7%) were qualitative, mixed methods, or included some interview component [19,25,26,33,37,44], 4 (11.1%) were nonrandomized cross-over studies [42,47,50,51], and 1 (2.8%) was a nonrandomized controlled study [48]. We present summaries of all articles in Multimedia Appendix 1.

In our review, we categorized articles into several themes to illustrate the diversity of the literature. We focus here on 5 themes for which we present narrative reviews of pertinent articles. First, while individual articles tended to focus on a particular pain type, conclusions from the literature are limited by the heterogeneity of chronic pain types. Second, intervention types were highly variable. Third, while most articles focused on pain outcomes, secondary and exploratory outcomes were highly variable. Fourth, the degree to which immersion was achieved was highly variable and not systematically explored in many articles. Fifth, side effect monitoring was limited in most research.

### Pain Types

While we expected to see variability in pain diagnoses because of our broad search terms, we found that the heterogeneity of pain types in the research to date limited external generalizability. In our literature search, 10 out of 36 (27.8%) articles focused on chronic pain, not otherwise specified [17,20,26,31,32,34,37,47,50,51]; 9 out of 36 (25%) focused on chronic back pain [22-24,28,29,35,44,45,49]; and 6 out of 36 (16.7%) focused on chronic regional pain syndrome [21,30,36,41,42,52]. Neuropathic pain was the focus of an additional 5 out of 36 (13.9%) articles, [18,36,38,40,48] but 3 were in the context of spinal cord injury [18,40,48]. Two out of 36 (5.6%) articles recruited patients with fibromyalgia [22,27]. Common pain types such as lower extremity osteoarthritis, diabetic neuropathy, abdominal pain, or noncardiac chest pain are absent from the primary literature, though it is possible many participants included in studies had multiple chronic pain types. There were no articles focusing on chronic overlapping chronic pain conditions, such as endometriosis, painful bladder syndrome, chronic fatigue syndrome, and irritable bowel syndrome.

### Intervention Types

To understand the breadth of literature, we included articles that ran the spectrum of VR intervention design. We found VR interventions were highly heterogeneous. The plurality (11, 30.6% studies) were on VR behavioral therapy interventions [17,22-26,34,35,38,43,45]. Among these 11 studies, behavioral therapies included related but disparate modalities, including mindfulness techniques (8 out of 11) [17,22-26,34,35] pain education (6 out of 11) [17,22-24,35,45], and hypnosis (2 out of 11) [38,43]. Another 10 (27.8%) studies immersed participants in noninteractive passive environments, such as landscapes or walks [18,19,26,32,33,40,46,47,50,51]. An additional 6 (16.7%) used interactive, and sometimes strenuous, games in VR to attempt to reduce chronic pain [27-29,31,44,45]. VR body swapping or body observation was common in studies of complex regional pain syndrome or neuropathic pain, and constituted an additional 7 (19.4%) studies [21,30,36,41,42,49,52]. VR neurofeedback and “commercially available” VR applications constituted categories with 1 (2.8%) study apiece [20,39].

### Outcomes

Almost all non-qualitative studies used pain scales, either numerical rating scales or visual analog scales, as outcome measures (30/32, 96.9% studies). Pain outcomes were not primary outcome measures in 4 studies, all of which were either primarily feasibility or usability studies [28,33,37,47]. Other outcomes varied widely. Changes in mood were assessed in 9 articles [17,18,22-24,33,39,43,52], and changes in sleep were assessed in 5 articles [22-24,39,52]. Changes in kinesophobia or changes in fear avoidance were assessed in 5 articles [27-29,45,46]. Physiological changes, such as heart rate variability or skin temperature, were assessed in a further 3 articles [42,50,51]. Physical activity, quality of life, and opioid use were assessed as outcomes in 2 [27,52], 1 [27], and 1 article, respectively [17].

### Immersion

Immersion, or the tendency for a participant to feel the illusion of reality within a VR simulation, was uncommonly assessed. Only 3 studies systematically assessed immersion in the VR world [17,40,45]. Presence, or the tendency to feel one is within a virtual environment, is often used interchangeably with immersion and was assessed in 3 articles [18,19,40]. An additional 2 studies assessed engagement in VR, a related concept, but 1 which relates more to adherence and satisfaction than immersion [20,32].

### Side Effect Monitoring

Despite common reports of side effects in commercial VR devices, side effects were variably assessed in studies. There was no reported assessment of side effects in 21 (58.3%) studies [17,18,21,27-31,34-36,38-46,48]. Among studies that systematically reported side effects, most were low frequency, low intensity, or not durable. Most side effects did not adversely affect participation, but 2 studies reported participants dropping out due to side effects (Figure 1) [26,31].

## Discussion

### Principal Findings

In this scoping review, we describe patterns in the current literature on VR interventions in chronic pain. We identified 36 articles that tested VR interventions through a variety of study designs. We reported 5 themes that encapsulate research to date: (1) pain types are highly heterogenous in the literature to date, (2) VR design was highly variable, (3) secondary and exploratory outcome choice was highly variable, (4) VR immersion was infrequently assessed, and (5) side effect monitoring was limited. These themes are somewhat related and have implications for conclusions to be generated from the research published to date. We provide some recommendations for future research studies.

### Heterogeneity Between Studies and Comparison to Previous Work

Studies were heterogeneous in many aspects, including type of VR intervention, patient population, and study design. In the literature we reviewed, even the term VR was used inconsistently and was even used to refer to nonimmersive modalities and platforms such as computers and projector screens. In developing our search strategy, we found that “virtual reality” was not a universal term and had to exclude non immersive devices from our sample of articles; this heterogeneity of study design was also encountered by one other scoping review [53]. The patient population was additionally highly variable between studies. Another scoping review of seven studies on VR-based mindfulness programs for chronic pain noted differences in populations and VR interventions between studies [15]. Such differences pose challenges in making generalizable VR recommendations for patients with chronic pain, as different VR treatments may only ameliorate certain pain types. This in turn, if left unchecked, may negatively affect the future generalizability and use of VR as a treatment for chronic pain.

There was significant heterogeneity in pain type among those receiving VR interventions. The existing literature on VR for the most common pain types is lacking. For example, it is difficult to find studies on previously mentioned common types such as lower extremity osteoarthritis, diabetic neuropathy, and abdominal pain. Instead, there have been many more studies on VR modalities focusing specifically on rarer types such as phantom limb pain and complex regional pain syndrome. This is likely because there already exist therapeutic elements backed by an evidence base (eg mirror therapy) in treating these painful conditions that can be directly incorporated into VR. Of note, VR behavioral management and VR therapies directly targeting kinesiophobia seem to have an emerging evidence base, stemming from their evidence toward treating relevant pain types in non-VR trials. For example, Lin and colleagues analyzed evidence for pain neuroscience education, a theoretical framework for educating and empowering the patient on his or her pain condition and management options [54]. They found evidence for pain neuroscience education in reducing pain and kinesiophobia in patients with chronic neck pain. Likewise, a recent review on cognitive behavioral therapy (CBT) and chronic pelvic pain has noted the efficacy of CBT in treating many conservative treatment-refractory pain types such as nociplastic pain disorders [55]. Research on employing CBT or CBT elements in VR interventions, and how best to do this without a live, skilled therapist, is a focus of current research, including our own ongoing clinical trials.

Ultimately, which VR interventions to offer which patients and when and how are important and significant questions for which current data is still insufficient. The insufficiency of current data to date was echoed in other recent scoping reviews of VR interventions [12,13,15,18,53,56]. A recent scoping review of 44 studies in chronic pain also concluded that while VR showed promise, the exploratory nature and heterogeneous design of most research limits generalized conclusions [13]. Further, another scoping review of 13 studies in back pain also found that research was highly variable and concluded the field was still nascent [12]. Future research should build on exploratory research conducted to date and should also include comparative effectiveness studies between VR intervention types to move the field forward. In addition, consistent, regular assessment of participant side effects is imperative to producing clear risk and benefit data for clinicians and patients in the future.

Relatedly, we found a lack of theoretical rigor in most studies of VR. Basing study design on theory can help elucidate mechanisms for how interventions work and can help researchers build more robust clinical trials. In particular, such a framework would include identifying and testing the various elements of the VR experience, isolating the aspects of VR that can lead to pain relief, and understanding whether there are mediation or moderation effects that influence the efficacy of VR interventions in chronic pain. For example, immersion is theorized to be a key target for how VR works on chronic pain [57-60]. Nonetheless, as we have shown, it was rarely systematically assessed. Different VR interventions can have markedly different effectiveness in generating immersion, owing to many factors: hardware weight and comfort, graphical fidelity, and the level of involvement and stimulation. Other important mediators, such as mood or fear of physical activity, are also infrequently assessed. Moderators, such as a participant’s comfort with technology and video games are also rarely assessed formally. No strong theoretical model yet exists to explain how VR interventions might work in chronic pain [61]; it is important that future research aims to develop and empirically test theoretical models to ensure generalizable knowledge is produced. In addition, and specifically, future studies should measure and compare levels of immersion between various VR types.

### Limitations

There were a few limitations to this scoping review. First, we searched 2 large databases, PubMed and CINAHL, for relevant articles because they constitute a significant portion of the clinical literature in chronic pain and we used a broad searching strategy with broad search terms, but it is possible we may have missed some manuscripts due to our search strategy. Future reviews can determine the utility of searching other comprehensive databases. Second, we included only English language articles in our initial search. This may have excluded data from other countries which may alter the conclusions drawn by our study. Future reviews can determine how non-English literature might differ from the English language articles included in this review. Third, we note that this article is unable to make a detailed examination of particular methodologies of VR interventions, partly because of a lack of data but also partly because of lack of comprehensive detail on VR methods in many of the articles reviewed. This is a limitation of the literature at large at this point, and it is very important for the reproducibility of research that there is transparency in research methods. Future VR interventional studies should include more descriptive details about the methodologies of the VR interventions used.

### Conclusions

In summary, we found the literature on VR in chronic pain to be highly variable and lacking theoretical rigor. While there is emerging evidence that supports VR use in a wide variety of health conditions including chronic pain, future research should focus on producing theoretically rigorous work that focuses on mechanisms, and transparency in research methods and that systematically assesses side effects in order to generate robust generalizable knowledge.

## Appendix

Multimedia Appendix 1 Summaries of analyzed articles (n=36).

Multimedia Appendix 2 Updated PRISMA ScR checklist.

## Abbreviations

CBT: cognitive behavioral therapy
MeSH: Medical Subject Headings
PRISMA-ScR: Preferred Reporting Items for Systematic Reviews and Meta-Analyses extension for Scoping Reviews
VR: Virtual reality

## Data Availability

The datasets generated during and/or analyzed during this study are available from the corresponding author on reasonable request.
