# Supplementary material for: Virtual Reality Interventions and Chronic Pain: Scoping Review
Source: J Med Internet Res. 2025 Feb 18;27:e59922. doi: 10.2196/59922 (PMC11888090; doi:10.2196/59922)
Supplement: Multimedia Appendix 1 [file jmir_v27i1e59922_app1.pdf]

This is a Multimedia Appendix to a full manuscript published in the J Med Internet Res. For full copyright and citation information see http://dx.doi.org/10.2196/jmir.59922

Table: Summaries of Analyzed Articles (n= 36)

Authors, Year, Country, Journal	Research Aim	Intervention & Control	Study Design	Participants	Adverse Event Reporting	Results
Abd-Elsayed et al. 2021[17]<br><br>United States, *Pain Ther*	To evaluate the effectiveness of the Harvard MedTech Vx Pain Relief Program on adult patients with acute and chronic pain.	Intervention: The Harvard MedTech Vx Pain Relief Program consisted of both home-based virtual reality therapy (VRT) and tailored behavioral therapy that was conducted through phone consultations. VRT lasted 90 days; participants were instructed to use Vx headset at least 1-2 times daily for 45 mins. VRT exercises includes experiences related to concepts such as knowledge of pain, distraction from pain, or meditation techniques. Behavioral therapy (30-60m each week) consisted of discussing progress with headset and goal-setting.	Retrospective single-arm cohort study. Primary outcome: VAS pain scores. Secondary outcomes: Change in opioid use, average time thinking about pain, anxiety awareness and level of immersion, perception that goals were achieved.	Population of interest:<br><br>Adult patients with acute or chronic pain. Total participants: 36. Average age 45, 16/36 were male.	No assessment of side effects reported.	Primary outcome: Pain reduction of 40% while using VR headset, statistically significant at each weekly session. Secondary outcomes: 69% of patients reported decrease of cessation of opioids. 72% reduction in average hours thinking about pain. 70% reduction in anxiety due to pain. Strong immersion and satisfaction. Strong improvements in goals related to activity, attention, and endurance.

Austin et al. 2021[18]<br><br>Australia, *Spinal Cord*	To determine whether 3D VR is more effective than a 2D screen-based device in reducing neuropathic pain in spinal cord injury	Intervention: 3D VR non-interactive experience called Nature Trek, delivered one time over 15 minutes. Control: 2D non-interactive viewing of Nature Trek on a laptop screen, delivered one time over 15 minutes.	Randomized cross-over study. Primary outcome: Numerical pain rating scale. Secondary outcomes: Change in Depression Anxiety Stress Scale, iGroup Presence Questionnair e.	Population of interest: People with spinal cord injury and chronic neuropathic pain. Total participants: 16. Average age 54.3. 100% male.	No assessment of side effects reported.	Primary outcome: 3D VR was associated with significantly greater reductions in pain intensity from baseline compared to 2D video. Standardized mean difference (Hedges' g) for pain reduction in 3D versus 3D was 0.80, which indicates a large effect. Secondary outcomes: No significant differences in mood between groups. Presence was associated with greater reduction in pain intensity.
Austin et al. 2022[19]<br><br>Australia, *Supportive Care in Cancer*	To evaluate the feasibility of recruitment for VR and data collection procedures and acceptability and comfort of VR devices in people receiving palliative care for cancer pain both in hospital and at home. Additionally, to gather preliminary data on whether a 3D VR device results in reductions in chronic pain compared to a 2D screen-based device.	Intervention: 3D VR non-interactive experience called Nature Trek, delivered one time over 15 minutes. Control: 2D non-interactive viewing of Nature Trek on a laptop screen, delivered one time over 15 minutes.	Randomized cross-over study. Primary outcome: Numerical pain rating scale. Secondary outcomes: Edmonton Symptom Assessment System, Australian modified Karnofsky Perfomance Status, iGroup Presence Questionnair e, Feasibility/ Qualitative semi-structured interview.	Population of interest: Palliative care patients with cancer inpatient or at home. Total participants: 14. Average age 71.1. 6/14 Female. 11/14 inpatient.	No specific VR side effects reported, though some reporting as part of normal assessments. No significant symptoms of nausea or cybersickness noted.	Primary outcome: 3D and 2D produced significant reductions in chronic pain immediately after both interventions. No significant difference between 3D and 2D. Secondary outcomes: Higher presence from 3D VR, and presence was associated with greater reductions in pain intensity. Significant reductions in drowsiness, shortness of breath, and improvement in wellbeing in both 3D and 2D. Semi-structured interviews noted high satisfaction.

Baker et al. 2022[20]<br><br>United States, *Pain Medicine*	To explore the feasibility of integrating VR into a chronic pain program, to explore whether VR reduced pain during treatment and was acceptable, and to assess adverse events.	Intervention: 10 minutes of "commercially available" VR programs for up to 6 visits, followed by participation in regular occupational therapy treatment.	Single-arm cohort study. Primary outcome: Numerical pain rating scale. Secondary outcomes: User Engagement Scale.	Population of interest: Patients from an outpatient occupational therapy program with chronic pain. Total participants: 29. Average age 42.6. 23/29 Female. 17/29 with chronic pain syndrome and 7/29 with fibromyalgia.	Common VR side effects reported. 2 ppts reported side effects.	Primary outcome: Large clinically significant reductions in pain during VR. More than 75% of participants achieved clinically important reductions in pain. Secondary outcome: High engagement in the VR intervention.
Chau et al. 2020[21]<br><br>United States, *Innov Clin Neurosci*	To explore the effects of therapeutic immersive virtual reality (VR) on pain in upper limb complex regional pain syndrome (CRPS).	Intervention: 10 VR therapy sessions (1-3/ week), each lasting 45 minutes-1 hr, that consisted of guided visualization exercises and interactions with the virtual environment using their virtual hands (washing hands, tossing paper airplane, assembling a sandwich, sorting dishware, arranging utensils).	Single-arm case series. Primary outcome: Short Form McGill Questionnaire. Secondary outcomes: VAS pain scores, Wong-Baker FACES score.	Population of interest: Outpatients with CRPS in at least one upper limb. Total participants: 8. Average age 45.4. 7/8 Female.	No assessment of side effects reported.	Primary outcome: Lower pain scores after VR during each session, but higher scores over time, reflecting higher pain. No statistical tests were performed. Secondary outcomes: Objective worsening in pain scores, but subjective improvement in symptoms and daily function.

Darnall et al. 2020[22]<br><br>United States, *JMIR Form Res*	To evaluate a skills-based VR behavioral medicine VR program for cLBP and fibromyalgia.	Intervention: VR therapy that teaches self-management skills based on CBT and mindfulness used in pain management. Delivered at home over 21-days; each session was 1-15 minutes. Control: Audio narrative content congruent with the VR intervention, with approximately two-thirds identical to the VR intervention. Adaptations made to one-third of audio content owing to lack of images in audio control. Delivered at home over 21-days; each session 1-15 minutes.	Randomized controlled trial. Primary outcome: Pain numerical rating scale overall last 24 hours. Secondary outcomes: Pain interference on activity, mood, sleep, and stress; Pain Catastrophizing scale, Pain Self-Efficacy Questionnaire, Patient Global Impression of Change, Satisfaction with Treatment.	Population of interest: Adults with chronic non-malignant low back pain or fibromyalgia. Total participants: 97 (analytic sample 74). Average age not reported; 43% 55+. 70% male. 66% white. 38% with High School education or less.	Adverse events were systematically assessed post-treatment. 24% reported some side effects, mostly at low frequency.	Primary outcome: Pain intensity decreased over time, with a steeper non-significant decline in VR versus audio groups. Secondary outcomes: Pain interference decreased more in the VR versus audio group. Pain-related interference on mood, sleep, and stress decreased more in the VR versus audio group. Pain interference decreased over time, but there was a non-significant difference by group over time. Pain self-efficacy differed over time, but not by group. 84% of VR group ppts noted their pain was improved, versus 62% in the audio group.

Garcia et al. 2021a[23]<br><br>United States, *Journal of Medical Internet Research*	To conduct a placebo controlled RCT in community-based individuals with CLBP to test therapeutic VR with chronic pain education and pain relief skills vs sham VR.	Intervention: EaseVRx program, an immersive pain relief skills VR program that teaches self-management skills based on CBT and mindfulness. Delivered at home, daily over 56 days; each session 1-15 minutes.<br><br>Control: Sham VR. 2D VR nature content. Delivered daily over 56 days; each session 1-15 minutes.	Randomized controlled trial. Primary outcome: DVPRS pain intensity. Secondary outcomes: DVPRS pain interference on activity, DVPRS pain interference on mood, DVPRS pain interference on sleep, DVPRS pain interference on stress, PROMIS physical function, PROMIS sleep disturbance.	Population of interest: Adults with self-reported non-malignant low back pain. Recruited online through organizations or through Facebook. Total participants: 179. Average age 51.5. 76.5% female. 90.5% white.	Adverse events were systematically assessed in post-treatment survey. 9.7% in EaseVRx group experienced nausea or motion sickness; 6.7% in ShamVR group. No participants reported AEs during treatment.	Primary outcome: EaseVRx showed significant treatment effect over time with large effect size and moderate clinical importance on pain interference. 65% of EaseVRx participants achieved 30% or more reduction in pain intensity, versus 40% in ShamVR group. Secondary outcome: Significant decrease by treatment over time with pain interference on activity. Significant decrease by treatment over time with pain interference on mood. Non-significant decrease by treatment over time with pain interference on sleep. Significant decrease by treatment over time with pain interference on stress. Significant increase in physical function by treatment over time. Significant decrease in sleep disturbance by treatment over time.

Garcia et al. 2021b[24]<br><br>United States, *The Journal of Pain*	The objective of the current report was to quantify treatment effects to post-treatment month 3 and describe durability of effects of VR on chronic pain studied in a prior study's 8-week home VR program.	Intervention: EaseVRx program, an immersive pain relief skills VR program that teaches self-management skills based on CBT and mindfulness. Delivered at home, daily over 56 days; each session 1-15 minutes.<br><br>Control: Sham VR. 2D VR nature content. Delivered daily over 56 days; each session 1-15 minutes.	Randomized controlled trial. Primary outcome: DVPRS pain intensity. Secondary outcomes: DVPRS pain interference on activity, DVPRS pain interference on mood, DVPRS pain interference on sleep, DVPRS pain interference on stress, PROMIS physical function, PROMIS sleep disturbance.	Population of interest: Adults with self-reported non-malignant low back pain. Recruited online through organizations or through Facebook. Total participants: 188. Average age 51.7. 77% female. 91% white.	Adverse events were systematically assessed and described in another paper published with primary outcome data.	Primary outcome: EaseVRx showed significant treatment effect over time compared to ShamVR on pain interference. 46.8% of EaseVRx participants achieved 30% or more reduction in pain intensity versus 31.2% in Sham VR arm. Secondary outcomes: Significant decrease by treatment over time with pain interference on activity. Non-significant decrease by treatment over time with pain interference on mood. Non-significant decrease by treatment over time with pain interference on sleep. Significant decrease by treatment over time with pain interference on stress. Significant increase in physical function by treatment over time. Significant decrease in sleep disturbance by treatment over time.

Garrett et al. 2020,[25]<br><br>Canada, *Heliyon*	To better understand experiences from participating in an ongoing randomized controlled trial of a daily VR-based self administered home therapy for one month.	Intervention: VR experimental group consisted of a randomized series of four interventions. Two are based on cognitive engagement and two are based on mindfulness meditation. Each of four interventions is 30 minutes per day for 6 days; total participation is 4 weeks. Control: Non-VR control of the same randomized series of four interventions described above.	Qualitative research; two focus groups (6-7 participants in each group). Topics addressed: Participants' experiences and perceptions of the use of VR. Groups lasted 60 minutes each.	Population of interest: Patients above 16 with current or past diagnosis of cancer and chronic pain enrolled in an RCT. Total participants: 12 in qualitative study. 6/12 male. Age range 37-73.	Adverse events were assessed in the interview guide. All participants reported motion sickness with one VR app that involved flying, with tolerance developing and no significant lasting effects.	Qualitative summary: 1) Experiences. Experiences were generally positive. Participants favored mindfulness exercises over cognitive engagement exercises. 2) Usability. Some physical limitations and discomfort (sitting, weight of headset). 3) Effects. Majority reported positive experiences. VR beneficial in their pain management. 4/12 found no benefits. Some enjoyed the relaxing experience while negative comments focused on frustration, depression, confusion, and fear. Motion sickness was common. Mixed reaction to how long effects lasted. 4) Mode of action. Most comments reflected that relaxation and distraction appeared to be most significant factors.

Garrett et al. 2017,[26]<br><br>Canada, *JMIR Medical Informatics*	To explore the effectiveness of at home VR on pain and to establish feasibility of VR interventions in chronic pain.	Intervention: VR therapy consisting of 4 different experiences over 4 weeks. Week 1: passive VR experiences where they traveled through VR environment. Week 2: mindfulness focused VR applications. Week 3: active exploratory VR environments. Week 4: active problem-solving experiences. Participants were instructed to use for 30 min on every other day.	Mixed methods case series. Primary outcome: Pain numerical rating score. Secondary outcomes: Brief Pain Inventory, Short Leeds Assessment of Neuropathic Symptoms and Signs, Cybersickness Reporting Form, Qualitative interviews at start and end of study.	Population of interest: Adult patients diagnosed with a chronic pain condition for at least 6 months. Total participants: 8. Average age 51. 6/8 female.	Systematically assessed during study. One participant dropped out of the study due to cybersickness. 5/8 participants who were retained reported cybersickness for at least one VR experience. One participant noted symptoms persisted for some time after experience.	Primary outcome: No significant effect on pre-post intervention pain intensity. Secondary outcomes: No significant differences in Brief Pain Inventory or neuropathic symptoms. Qualitative summary: 1) Interactive sessions were favored over passive. 2) Distraction was cited as a reason for pain relief but this did not persist. 3) Frustrating user experience limited applicability. 4) Cybersickness was common.
Gulsen et al. 2020,[27]<br><br>Turkey, *Assistive Technology*	To evaluate the effects of fully immersive VR treatment combined with exercise training in fibromyalgia patients.	Intervention: Exercise + IVR (Immersive VR): 30 mins of aerobic training and 30 minutes of pilates plus 20 mins of IVR tx twice per week x 8 weeks. IVR included Kinect and Oculus VR football games and dungeon game. Control: Exercise: 30 mins of aerobic and 30 minutes of pilates twice per week x 8 weeks.	Randomized controlled trial. Primary outcome: Pain VAS. Secondary outcomes: Modified Sensory Organization Tests (for balance), Tampa Scale of Kinesiophobia, Fibromyalgia Impact Questionnaire, Fatigue Severity Scale, International Physical Activity Questionnaire, Health-related quality of life.	Population of interest: Adult patients with fibromyalgia. Total participants: 16. Average age in IVR group 46.5, average age in Exercise control group 38.5. 100% female.	No assessment of side effects reported.	Primary outcome: Exercise + IVR showed more significant improvement over time in pain scores compared to Exercise group. Secondary outcomes: No difference in balance. Significant improvements in exercise + IVR (vs exercise) in kinesophobia, Fibromyalgia impact, fatigue severity, physical activity, and mental component of quality of life.

Hennessy et al. 2019,[28]<br><br>United States, *The Journal of Pain (abstract)*	To assess feasibility of a VR graded exposure intervention for chronic lower back pain.	Intervention: VR gaming modules while on a self-driven treadmill. Users walk, reach, crouch to accomplish game objectives. 3 sessions over 1 week.	Single-arm cohort study. Primary outcome: Feasibility of using VR games. Secondary outcomes: Acceptability, pain intensity, Kinesiophobia.	Population of interest: Individuals with chronic low back pain. Total participants: 13. 8/13 female.	No assessment of side effects reported.	Primary outcome: High feasibility with high retention (12/13 completed study). Secondary outcomes: Participants viewed VR modules as acceptable. Pain intensity and kinesophobia did not change over the course of the study.
Hennessy et al. 2020,[29]<br><br>United States, *JMIR Serious Games*	To determine content validity of VR application and to determine the feasibility of persons with cLBP performing locomotion-enabled physical activities.	Intervention: VR application, Lucid, to practice real-world movement tasks along the spectrum of activities (reaching, bending, long lasting loads) in games that required different levels of each movement task to complete. 6 3-min modules, 3x/week for 1 week	Single-arm cohort study. Outcomes: Content validity of VR application, acceptability and usability, pain and pain-related fear.	Population of interest: Patients with low back pain for >3 months and high pain related fear. Total participants: 12. Average age 54.3. 8/12 female. 100% Black.	No assessment of side effects reported.	Outcomes: Higher avoidance, expected pain, and expected concern to the Lucid sessions that are designed to be more challenging. 92% of participants rated exercises as acceptable. 75% reported the system was usable. No changes to pain or pain-related fear.
Jeon et al. 2014,[30]<br><br>Korea, *Cyberpsychology, Behavior, and Social Networking*	To determine if virtual body swapping for patients with chronic regional pain syndrome type 1 (CRPS) is beneficial.	Intervention: VR video meant to evoke virtual body swapping illusion. Participants asked to act out maneuvers. 3 minute and 20 sec video, filmed from first person perspective. Control: VR video without being asked to act out maneuvers.	Randomized controlled trial. Primary outcome: Pain numerical rating score. Secondary outcomes: Modified Body Perception Disturbance Questionnaire, Perception of virtual body's movement as own body.	Population of interest: Patients with Chronic Regional Pain Syndrome CRPS type 1. Total participants: 10. Average age 39.3. 100% male.	No assessment of side effects reported.	Primary outcome: No significant difference between groups in pain intensity. Secondary outcomes: Significant improvement in body perception disturbance in intervention vs control. Treatment group experienced greater virtual body swapping illusion than the control group.

Jin et al. 2016,[31]<br><br>Canada, *Studies in Health Technology and Informatics*	To determine if an immersive VR game can be effective for chronic pain management.	Intervention: VR interactive game, Cryoslide, a game where you slide in an icy world and shoot snowballs at creatures. 10 minute duration. Control: Pain distracting activities, such as meditating, reading, mobile games, audiobooks. 10 minute duration.	Randomized crossover study. Primary outcome: Pain VAS. Secondary outcomes: Time spent thinking about pain.	Population of interest: Subjects recruited with chronic pain from a complex pain clinic. Total participants: 20. Age range 30-75. 16/20 female.	No assessment of side effects reported. 3 dropouts due to time constraints or nausea.	Primary outcome: Significant 36.7% reduction in pain intensity during the intervention, but no difference after the intervention. Secondary outcome: VR intervention group more likely to report "reduction in time thinking about pain," "losing track of time," non significantly lower "thinking unrelated things" or "thinking inwardly."
Jones et al. 2016,[32]<br><br>United States, *PLoS ONE*	To determine the impact of a brief VR session on the experience of pain in patients with chronic pain conditions.	Intervention: 5 minutes of engagement in VR experience called COOL!, which is an "Interactive journey through a fully immersive VR fantasy landscape."	Single-arm cohort study. Primary outcome: Pain numerical rating scale. Secondary outcomes: Engagement in VR, side effects.	Population of interest: Adults with chronic pain. Total participants: 30. Average age 50. 67% female. 96% white.	Systematically assessed during study. One participant reported mild nausea that did not prevent participation.	Primary outcome: Significant 33% reduction in pain. 33% of participants reported 100% pain relief while doing the session. Secondary outcomes: High engagement in VR world. Limited side effects.
Kelleher et al. 2022,[33]<br><br>United States, *Palliative and Supportive Care*	To examine feasibility, acceptability, safety and impact of a 30-minute VR experience for reducing pain and pain related symptoms in patients with advanced colorectal cancer.	Intervention: Single, 30 minute VR session of an underwater/sea environment.	Single-arm cohort study. Primary outcomes: Feasibility, acceptability, and safety. Secondary outcomes: BPI pain severity, Mood VAS, Pain Catastrophizing Questionnaire, Chronic Pain Self-Efficacy Scale, qualitative interview.	Population of interest: Patients with stage 4 colorectal cancer and moderate to severe pain. Total participants: 20. Average age 56.7. 14/20 male. 15/20 white.	Systematically assessed during study. All participants completed VR without self-report of significant side effects. One participant had self-limited mild dizziness.	Primary outcomes: Recruitment accrual was strong, with 20 participants recruited in about 6 months. All participants completed the intervention. VR found to be highly acceptable. Secondary outcomes: No statistical hypothesis testing was performed. Pain decreased, tension decreased, stress decreased, Anxiety decreased, relaxation increased, and mood improved. In qual study: Easy to use and comfortable, immersed. Majority preferred home use

Liu et al. 2021,[34]<br><br>United States, *Alternative Therapies*	To examine the efficacy of VR-guided meditation in US veterans to facilitate meditation and relaxation practice.	Intervention: One 10-minute VR-guided Zen meditation using Guided Meditation VR application; script focused on effortless breathing and mindfulness meditation with six backgrounds selected by participants.	Single-arm cohort study. Primary outcome: Pain numerical rating scale. Secondary outcomes: Stress numerical rating scale, resting-state BP and HR, experiences and attitudes.	Population of interest: Veteran patients with chronic headaches or other chronic pain. Total participants: 31. Average age 55.2. 93.5% male.	No assessment of side effects reported.	Primary outcome: Pain significantly reduced post intervention with small-to-medium effect size. Secondary outcome: Stress significantly reduced post intervention with medium effect size. Systolic and diastolic BPs significantly reduced post intervention with small-to-medium effect size. HR significantly reduced post intervention with small effect size. VR was acceptable and enjoyable to patients.
Louw et al. 2019,[35]<br><br>United States, *J of Physiotherapy Pain Association*	To describe how VR-delivered pain neuroscience education can lead to reductions in pain catastrophization.	Intervention: Three VR sessions using BehaVR: pain neuroscience education, breathing exercises, mindfulness. Sessions lasted 15-26 minutes and took place weekly.	Case study. Outcomes: Pain numerical rating scale, Neck Disability Index, Pain Catastrophizing Scale, formal measures of pressure pain thersholds of neck.	Population of interest: One 18-year-old participant with history of MVA and subsequent chronic headaches and neck and upper back pain.	No assessment of side effects reported.	Outcomes: Neck pain improved after each session. Neck disability improved 29%. Pain catastrophizing improved over 50%. Pressure pain thresholds decreased for the neck but increased for the low back.

Matamala-Gomez et al. 2019,[36]<br><br>Spain, *The Journal of Pain*	To determine how virtual arm characteristics can modulate pain ratings in patients with chronic pain due to Chronic Regional Pain Syndrome (CRPS) and Peripheral Nerve Injury (PNI).	Intervention: VR session with life sized virtual body, with instructions to position arm where virtual arm was. This was followed by a phase in which 4 different representations of the arm were displayed in random order, all related to how transparent the virtual arm was. Next, participants were exposed to 3 different size representations of the virtual arm, again presented in random order. Each representation lasted 45 seconds. Total time for session was 55 minutes.	Randomized cross-over study. Primary outcome: Pain intensity numerical rating scale, performed after each representati on. Secondary outcome: Ownership of virtual hand.	Population of interest: Patients with neuropathic chronic pain with CRPS type 1 or with peripheral nerve injury in the upper limb. Total participants: 19 (CRPS 9; peripheral nerve injury 10). Average age CRPS 43.8, PNI 52.7. CRPS 7/9 female, PNI 7/10 female.	No assessment of side effects reported.	Primary outcome: All 7 conditions reduced pain ratings by half. Increasing transparency decreased pain in CRPS but increased pain in PNI. Secondary outcomes: Participants expressed ownership over virtual arm.
Nakad and Rakel 2019, [37]<br><br>United States, *Innovation in Aging (abstract)*	To determine attitudes of older adults to VR distraction therapy for chronic pain using mixed methods techniques.	Intervention: 2 VR simulations, active and passive, lasting 10 minutes each.	Single-arm cohort study. Primary outcome: Treatment acceptability. Secondary outcomes: Usability and side effects. Qualitative study of experiences also performed.	Population of interest: Older adults with chronic musculoskeletal pain. Total participants: 21.	Assessment of side effects reported. 14% of participants experienced moderate to severe side effects, but these are not outlined.	Primary outcome: Treatment acceptability was high (32.5/40). Secondary outcomes: Usability scores were mediocre (62.9/100). Qualitative analysis demonstrated VR was an enjoyable distraction to pain.
Oneal et al. 2008,[38]<br><br>United States, *Int J Clin Exp Hypn*	To describe a case of a patient with upper extremity neuropathic pain after use of VR hypnosis for 6 months.	Intervention: 33 Virtual Reality Hypnosis sessions over 6 months. Sessions in VR included audio recording of hypnotic induction, suggestions for pain relief, then immersive 3D world.	Case study. Outcomes: Pain intensity numerical rating scale, pain unpleasantn ess numerical rating scale.	Population of interest: One 36-year-old patient with upper extremity neuropathic pain after car accident.	No assessment of side effects reported.	Outcomes: VRH did not lead to significantly different pain intensity or pain unpleasantness at post treatment or 1 month follow-up. VRH led to 36% reduction in pain intensity immediately after VR treatment, 33% reduction in pain unpleasantness.

Orakpo et al. 2021[39]<br><br>United States, *Frontiers in Psychiatry*	To test VR with neurofeedback for sustained analgesia for centralized pain.	Intervention: 20 sessions of VR-neurofeedback with EEG monitoring, two sessions weekly for 10 weeks.	Case study. Outcomes: Pain intensity, pain interference, pain related anxiety, sleep deprivation due to pain, pain related stress, pain related fatigue, depression due to pain.	Population of interest: One 55-year old woman with cervical stenosis with radiculopathy, sciatica with persistent right sided shoulder and neck pain.	No assessment of side effects reported.	Outcomes: 40% decrease in pain intensity. 40% decrease in Pain interference with activities of daily living, 50% decrease in pain interference with instrumental activities of daily living, 50% in Pain related anxiety, 10% decrease in Sleep deprivation, 40% decrease in pain related stress. Pain intensity decrease was durable, with 80% decrease in pain intensity at 1 year follow-up.
Putrino et al. 2021,[40]<br><br>United States, *International Journal of Environmental Research and Public Health*	To investigate the effect of 2 VR protocols (somatic vs scenery virtual environments) on pain intensity in people with spinal cord injury.	Intervention: Two VR environments, scenery (passive nature experiences) and somatic (upper and lower extremity movements), each VR session lasting 10 minutes. Presented in random order; participant allowed to take as much time as necessary for washout afterwards.	Randomized cross-over study. Primary outcome: Neuropathic pain numerical rating scale. Secondary outcomes: Immersive Tendencies Questionnaire, UQO-PQ Presence Questionnaire.	Population of interest: Adult persons with chronic neuropathic pain after spinal cord injury. Total participants: 8. Average age 55. 50% female.	No assessment of side effects reported.	Primary outcome: Significant reduction in pain in both VR environments. No significant difference between VR environments. Secondary outcomes: Greater immersion score in scenery intervention, greater decrease in pain; this was not true in the somatic intervention. Presence showed no significant correlations.
Solca et al. 2021,[41]<br><br>United States, *PAIN*	To test an integrated spinal cord stimulation and VR method that shows patients visual illumination of a circumscribed region on the patients legs corresponding to stimulated sections.	Intervention: Spinal cord stimulation (SCS) enhanced by VR. Participants had VR congruent illumination of leg as SCS was stimulated. 2 sessions at 24 hours interval. Control: Two controls: incongruent illumination and no illumination.	Randomized cross-over study. Primary outcome: Pain intensity visual analog scale. Secondary outcome: Embodiment in VR.	Population of interest: Patients with SCS implants for chronic leg pain due to Complex Regional Pain Syndrome or Failed Back Surgery Syndrome. Total participants: 15. Average age 47.7. 10/15 male.	No assessment of side effects reported.	Primary outcome: Significantly larger analgesic effect during congruent VR sessions than 2 control conditions; incongruent also had larger effect than VR alone. Average pain levels decreased 44% from baseline. Secondary outcome: SCS enhanced by VR induced changes in leg embodiment.

Solca et al. 2018,[42]<br><br>Switzerland, *Neurology*	To test the effect of heartbeat enhanced VR in patients with complex regional pain syndrome (CRPS).	Intervention: VR virtual hand on a table matched to laterality of disease in the participant, sex, and skin color. VR has synchronous flashing of virtual hand with heartbeat. One experiment, repeated 3 times consecutively. Control: VR virtual hand as above, but asynchronous flashing with heartbeat.	Cross-over study. Primary outcome: Pain intensity visual analog scale. Secondary outcomes: Grip strength, heart rate variability, embodiment in VR.	Population of interest: Patients with upper limb CRPS after upper limb trauma or stroke. Total participants: 24. Age range 23-73. 14/24 female.	No assessment of side effects reported.	Primary outcome: Decrease in pain intensity after synchronous compared asynchronous blocks. Secondary outcomes: Grip strength increased more after synchronous blocks compared to asynchronous blocks. Heart rate variability increased during synchronous blocks compared to asynchronous blocks. Embodiment was high, and similar in synchronous vs asynchronous blocks.
Soltani et al. 2011,[43]<br><br>United States, *Contemp Hypn Integr Ther*	To describe a case of a patient who used VR hypnosis for pain from hidradenitis.	Intervention: 30 minutes of VR hypnosis, which consisted of an audio recording and a trip through a VR environment followed by hypnotic suggestions, twice over 2 days. Each session 30 minutes.	Case study. Outcomes: Pain intensity, anxiety, time spent thinking about pain.	Population of interest: One hospitalized 55 year old African American female with gluteal hidradenitis.	No assessment of side effects reported.	Outcomes: Pain decreased by 70%, anxiety by 100%, time thinking about pain by 89%. However, opioid analgesics also increased between day 1 and day 2.
Stamm et al. 2020,[44]<br><br>Germany, *Journal of Neuroengineering and Rehabilitation*	To determine requirements for a VR game that could be used to supplement multimodal pain therapy for patients with chronic back pain.	Intervention: After semi-structured interviews, participants were asked to demo two VR application prototypes: 1) teaching posture through game mechanics, and 2) experiencing a passive tour of an immersive virtual environment.	Qualitative study. Outcomes: Semi-structured qualitative interview with patients, focus groups with physiotherapists and psychotherapists.	Population of interest: Older adults over 65 with chronic back pain. Total participants: 6. Average age 75.9.	No assessment of side effects reported.	Outcomes: Recommendations included an individual briefing for the system with sufficient time for instruction. Also emphasized, among other recommendations, was user-friendliness, and age-appropriate feedback.

Stamm et al. 2022,[45]<br><br>Germany, *Virtual Reality*	To test an active VR exergame for older patients with chronic back pain.	Intervention: VR exergame with movement therapy and psychoeducation for four weeks, 3 appointments per week lasting about 30 minutes each. Control: Chair based group exercises, four week movement therapy with seated exercises and psychoeducation in a group setting, 3 appointments per week lasting about 30 minutes each.	Randomized controlled trial. Primary outcome: Composite of pain intensity, changes in functional capacities, changes in fear avoidance, changes in maximum trunk muscle strength, and muscular imbalance. Secondary outcomes: SF-12 Health Survey, Immersion of VR system.	Population of interest: Older adults with chronic back pain. Total participants: 22. Average age 75. 14/22 female.	No assessment of side effects reported.	Primary outcome: Both groups showed a reduction in pain intensity, but there was no difference between VR group and chair-based group. Significant improvement in functional capacity in the VR group. No difference in fear avoidance in either group. Secondary outcomes: No change in general physical or mental health in VR group. High ratings for immersion of VR system.
Tejera et al. 2020,[46]<br><br>Spain, *International Journal of Environmental Research and Public Health*	To compare the effects of VR versus exercise on pain intensity in patients with non-specific chronic neck pain.	Intervention: Two VR applications: 1) Fulldive VR, simulating living room of house, utilizing tilting movements of the neck; 2) VR Ocean, where neck movements must be integrated in passive environment. Two treatment sessions per week for 4 weeks. Control: Neck exercises: flexion exercises, extension exercises, rotation and tilt exercises.	Randomized controlled trial. Primary outcome: Pain intensity visual analog scale, conditioned pain modulation. Secondary outcomes: Neck range of motion, neck disability, kinesiophobi [illegible]	Population of interest: Patients with non-specific chronic neck pain. Total participants: 44. Average age 29.7. 23/44 female.	No assessment of side effects reported.	Primary outcome: No significant differences between groups in pain intensity, conditioned pain modulation. Secondary outcomes: VR group led to lower pain kinesiophobia scores compared to control. Range of motion, neck disability, fear avoidance all no significant differences between groups.

Tong et al. 2016,[47]<br><br>Canada, *Stud Health Technol Inform*	To assess the usability of a VR and an enhanced desktop display to determine if these are suitable in patients with chronic pain.	Intervention: VR Meditative Walk, which involves walking through an environment with a forest, blue sky, small ponds and mountains." Intervention lasted 10 minutes and utilized head mounted display (HMD). Control: Meditative Walk app, but delivered through DeepStream 3D, a desktop display with 3D capabilities.	Cross-over usability study. Outcome: Simulator sickness questionnair e.	Population of interest: Patients from a pain clinic with chronic pain. Total participants: 20. Age range 20-70. 13/20 female.	Systematically assessed during study. Oculomotor symptoms, nausea, and physical discomfort tended to predominate, and were worse in the HMD compared to desktop display.	Outcome: HMD had higher Simulator Sickness Questionnaire scores than desktop display. Patients enjoyed experience of larger field of view afforded by HMD versus desktop display HMD caused more discomfort due to weight.
Trost et al. 2022,[48]<br><br>United States, *PAIN*	To test feasibility and preliminary efficacy of the first fully immersive spatially tracked virtual reality walking interface for patients with neuropathic pain due to spinal cord injury.	Intervention: Interactive VRWalk: arm movement tracked and translated to leg movements in an immersive environment with compensation for exploratory behaviors; 10 successive days of intervention with 2 sessions per day, at least 4 hours apart. Control: Passive control with no control over virtual gait.	Non-randomized controlled trial. Primary outcome: Pain intensity numerical rating score. Secondary outcomes: Last week pain intensity numerical rating score, neuropathic pain quality, pain interference.	Population of interest: Participants with spinal cord injury and neuropathic pain. Total participants: 27. Average age 42.5. 22/27 male. 17/27 Black.	No assessment of side effects reported.	Primary outcome: Both groups experienced declines in current neuropathic pain intensity with no difference between groups. Secondary outcomes: Significant decrease in average neuropathic pain in interactive group vs passive group. Significant decrease in neuropathic pain interference in interactive group vs passive group. Interventions found to be highly acceptable.
Trujillo et al. 2020,[49]<br><br>United States, *Journal of Pain Research*	To assess the feasibility of embodiment in VR for decreasing pain intensity and catastrophizin g of chronic lower back pain.	Intervention: 7 sessions of KVET, a 30–45-minute session of exercises in an embodied VR experience, based in principles of graded motor imagery. Observe virtual avatar performing a task, visualize it themselves, then perform it.	Case series. Outcomes: Pain intensity visual analog scale, pain catastrophizi ng.	Population of interest: Patients with chronic low back pain of at least six months. Total participants: 2. 37M and 64M.	Systematically assessed during study. Neither patient reported side effects.	Outcomes: Significant improvement in pain intensity after single sessions of KVET for both participants. Pain catastrophizing showed decrease from pre to post sessions.

Wiederhold 2014a,[50]<br><br>United States, *Cyberpsychology, Behavior, and Social Networking*	To test mobile phone displays to deliver pain distraction VR.	Intervention: Virtual environments on a mobile phone display. Control: Virtual environments in a head mounted display (HMD).	Cross-over study. Primary outcome: Pain intensity numerical rating scale and visual analog scale. Secondary outcomes: Physiological activity.	Population of interest: Patients with non-cancer chronic pain. Total participants: 31.	Unclear if systematically assessed. "No cybersickness" was reported.	Primary outcome: HMD significantly decreased pain intensity greater than mobile phone. Secondary outcome: Heart rate was decreased in HMD and mobile phone conditions, but this did not reach statistical significance.
Wiederhold 2014b,[51]<br><br>United States, *Cyberpsychology, Behavior, and Social Networking*	To investigate the efficacy of an interactive VR virtual environment on pain.	Intervention: VR exposure session, 14 minutes of pleasant and relaxing scenes with natural habitats. Control: Non-VR pain focus session, not fully described here.	Cross-over study. Outcomes: Pain intensity numerical rating system, skin temperature, physiological activity.	Population of interest: Patients with chronic pain. Total participants: 40 (6 in a pilot, 34 in an additional study).	Systematically assessed during study. Very low side effects reported. No serious side effects.	Outcomes: Decreased pain in VR exposure group compared to pain focus group. Increase in overall mean temperature in VR exposure group compared to pain focus group in pilot sample. In experimental sample, significant decrease in heart rate in VR exposure group vs pain focus group.
Won et al. 2021,[52]<br><br>United States, *Journal of Medical Internet Research*	To pilot test the feasibility of an open source VR mirror module in patients with upper limb chronic regional pain syndrome.	Intervention: Weekly sessions (min 4) of immersive VR with optional 5th visit. Participants experienced a virtual environment that consisted of a modification of mirror therapy, whereby affected arm was controlled by the unaffected arm. VR sessions were movement and goal oriented to contact objects in the midline.	Single-arm cohort study. Outcomes: Pain intensity, physical activity, mood, quality of sleep.	Population of interest: Patients with upper limb unilateral chronic regional pain syndrome. Total participants: 9. Average age 44. 6/9 female. 7/9 white.	Systematically assessed during study. 7/9 reported no symptoms of cybersickness. 2/9 reported slight symptoms.	Outcomes: No statistically significant differences over time on pain, physical activity, mood, quality of sleep.
